# Alpha protons as NMR probes in deuterated proteins

**DOI:** 10.1007/s10858-019-00230-y

**Published:** 2019-02-14

**Authors:** Kumar Tekwani Movellan, Eszter E. Najbauer, Supriya Pratihar, Michele Salvi, Karin Giller, Stefan Becker, Loren B. Andreas

**Affiliations:** Department of NMR Based Structural Biology, Max Planck Institute for Biophysical Chemistry, Am Fassberg 11, Göttingen, Germany

**Keywords:** Isotopic labeling, NMR, Membrane proteins, Structural restraints, Transamination, l-Amino acid oxidase

## Abstract

We describe a new labeling method that allows for full protonation at the backbone Hα position, maintaining protein side chains with a high level of deuteration. We refer to the method as alpha proton exchange by transamination (α-PET) since it relies on transaminase activity demonstrated here using *Escherichia coli* expression. We show that α-PET labeling is particularly useful in improving structural characterization of solid proteins by introduction of an additional proton reporter, while eliminating many strong dipolar couplings. The approach benefits from the high sensitivity associated with 1.3 mm samples, more abundant information including Hα resonances, and the narrow proton linewidths encountered for highly deuterated proteins. The labeling strategy solves amide proton exchange problems commonly encountered for membrane proteins when using perdeuteration and backexchange protocols, allowing access to alpha and all amide protons including those in exchange-protected regions. The incorporation of Hα protons provides new insights, as the close Hα–Hα and Hα–H^N^ contacts present in β-sheets become accessible, improving the chance to determine the protein structure as compared with H^N^–H^N^ contacts alone. Protonation of the Hα position higher than 90% is achieved for Ile, Leu, Phe, Tyr, Met, Val, Ala, Gln, Asn, Thr, Ser, Glu, Asp even though LAAO is only active at this degree for Ile, Leu, Phe, Tyr, Trp, Met. Additionally, the glycine methylene carbon is labeled preferentially with a single deuteron, allowing stereospecific assignment of glycine alpha protons. In solution, we show that the high deuteration level dramatically reduces R_2_ relaxation rates, which is beneficial for the study of large proteins and protein dynamics. We demonstrate the method using two model systems, as well as a 32 kDa membrane protein, hVDAC1, showing the applicability of the method to study membrane proteins.

**Electronic supplementary material:**

The online version of this article (10.1007/s10858-019-00230-y) contains supplementary material, which is available to authorized users.

## Introduction

The study of proteins by nuclear magnetic resonance (NMR) has been continuously evolving to improve sensitivity in order to resolve signals in multidimensional spectra, which serve as the basis for studies of structure and dynamics. For large proteins that tumble slowly in solution, as well as for proteins in the solid state, a high level of deuteration with introduction of selective protons is used to improve proton relaxation, and therefore narrow lines, by elimination of strong proton–proton dipolar couplings.

Proton detected magic-angle spinning (MAS) NMR studies have employed different combinations of spinning frequency and deuteration to optimize sensitivity and resolution (Andreas et al. 2015; Zhang et al. 2015; Wang and Ladizhansky 2014; Brown 2012; Chevelkov et al. 2006; Zhou et al. 2007; Lewandowski et al. 2011; Akbey et al. 2010). Currently, many applications of proton detected MAS NMR are applied at about 60 kHz with 1.3 mm rotors, a spinning frequency that for fully protonated samples is not enough to average the strong network of^1^H–^1^H dipolar couplings. This results in proton line broadening and about 200–300 Hz proton linewidths (Andreas et al. 2015). The advantage of this spinning frequency is that narrow lines are observed at high sensitivity when selected sites are labeled to 100% incorporation of protons, while others are deuterated.

In the most straightforward approach, *Escherichia coli* expression in D_2_O is followed by exchange with H_2_O/D_2_O to produce a protein with a specific protonation level at amides, and perdeuteration at non-exchangeable sites (Chevelkov et al. 2006; Akbey et al. 2010; Lemaster 1990). A protonation level of 100% at amide positions results in high resolution when using 40–60 kHz MAS in microcrystalline samples, (Lewandowski et al. 2011) enabling structure determination based on backbone resonances (Zhou et al. 2007).

Accessing aliphatic protons is still an area of active development. Sidechain protons can be selectively introduced using metabolic precursors, which has the advantage that a single isotopomer is typically present and deuterium isotope shifts do not result in broadening, even at 100% protonation of the selected sites. In various ways, methyl groups of I, L, V, T, A can be incorporated (Tugarinov et al. 2003; Isaacson et al. 2007; Velyvis et al. 2012). Exquisite control of labeling can be afforded using a synthetic approach known as SAIL labeling, with the only downside being the high cost, which has typically restricted applications to labeling only selected amino acid types in cell free expression systems (Kainosho et al. 2006). For fully protonated samples, high magnetic fields and very fast MAS ~ 100 kHz are required to sufficiently narrow proton resonances. At lower spinning frequencies, deuteration is still required, and approaches to resolve aliphatic protons involve using mixtures of H_2_O and D_2_O during protein expression, along with combinations of protonated or deuterated carbon sources such as glycerol or glucose (Lemaster 1990; Asami et al. 2010). While utilizing the residual protons in perdeuterated samples results in exquisite spectra (Agarwal and Reif 2008), the low labeling level severely limits the ability to measure proton–proton distances. At higher proton concentrations, the carbon resonances are broadened due to deuterium isotope shifts (Asami et al. 2012). Although this is a small effect for Hα, since only one proton is directly attached, it would still be desirable to limit the protons introduced in the sidechains, since such protons broaden the alpha resonance, and are a magnetization sink during proton–proton transfer.

We therefore sought a strategy that would allow labeling of alpha protons in *E. coli* at a cost that allows widespread adoption of the approach for structure determination and dynamics investigations. Previously, an approach for Hα labeling was introduced, where the proton was chemically exchanged in a deuterated amino acid mixture, producing a D/L mixture of amino acids, the L portion of which can be utilized directly by bacteria (Yamazaki et al. 1997). Although this previous method was successful, the alpha proton incorporation level was a problem for several amino acids, and serine and threonine were lost during the acetylation and deacetylation reaction. d-amino acids may also inhibit bacterial growth at high concentrations (Hishinuma et al. 1969; Bardaweel 2014). It was also noted that during growth on deuterated amino acid media (O’Brien et al. 2018; Fiaux et al. 2004), exchange of amide moieties occurs, but results in only 10–50% incorporation of alpha protons for hydrophobic residues (Löhr et al. 2003).

**Fig. 1:**
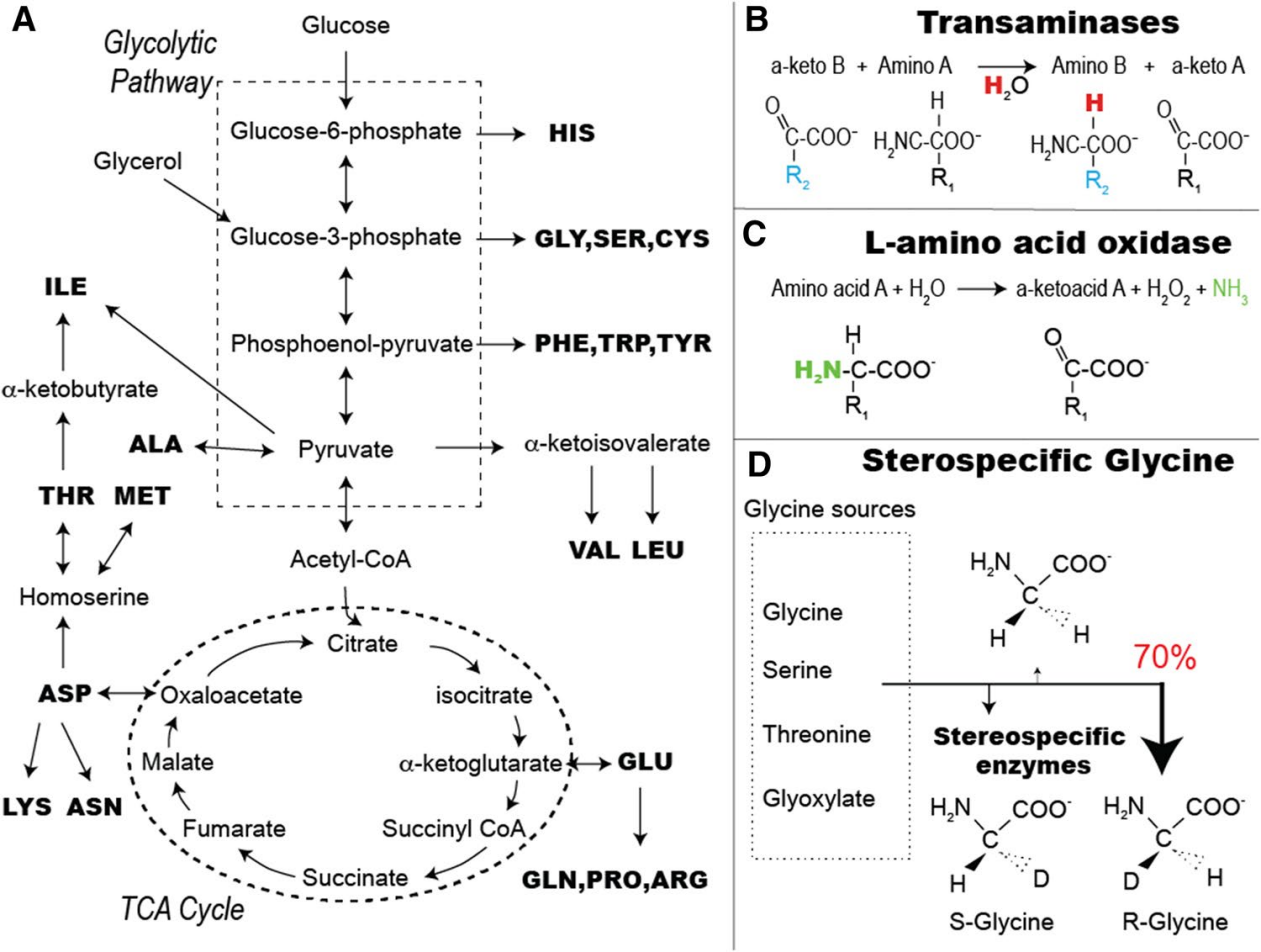
Amino acid metabolic pathways and the different enzymatic stages of the α-PET labeling method. The metabolic pathways of the TCA cycle are shown in **a**. In**b**, the transamination reaction is shown, which is the main route for Hα incorporation. In **c**, the generation of α-keto acids from amino acids by the enzyme LAAO is shown. **d** Shows the main biosynthesis pathways of glycine with the observed stereospecific labeling

We show an alternative approach that results in up to 100% Hα incorporation by supplying keto acids. The keto acids are converted by *E. coli* transaminases to the respective amino acids, while adding a proton at the alpha position from the water pool. This avoids any problems due to racemic amino acid mixtures, since the correct l-amino acids are generated enzymatically. The major pathways of amino acid synthesis from glucose and glycerol carbon sources are depicted in Fig. 1a for *E. coli*. Keto acids are often the direct precursor to an amino acid, indicating that provided a source of keto acids, protons can be introduced via transaminase activity (Fig. 1b), a method hereafter referred to as ‘alpha proton exchange by transamination’ (α-PET). This method, as with any where the growth medium is based on H_2_O, results in protonation of the amide position during protein expression, such that both the alpha and amide positions of the protein are protonated.

Some transaminases are amino acid-specific, like the glutamate–pyruvate aminotransferase that transfers the NH_3_ from glutamate to pyruvate forming alanine and α-ketoglutarate (Kim et al. 2010). Others are less specific for their substrate, such as branched-chain-amino-acid transaminase (BCAT) involved in leucine, isoleucine and valine anabolism (Rudman and Meister 1953). *E. coli* has a high diversity of such transaminase enzymes, resulting in effective labeling for the majority of residue types.

We generated keto acids by l-amino acid oxidase (LAAO) treatment of a commercial growth medium that is primarily comprised of ^2^H,^13^C, ^15^N-amino acids. LAAO enzymes are found in many organisms (Hossain et al. 2014), with different specificity for the substrate amino acids (Nuutinen et al. 2012; Sun et al. 2010). We chose as the enzyme source a crude snake venom containing LAAO, which can be applied directly to the commercial growth medium (Fig. 1c). Additional metabolic pathways might also be important, for example, we observed stereospecific labeling of glycine, which can occur through transaminase, but also by conversion of serine and threonine (Fig. 1d).

Here we show successful introduction of alpha protons for 13 amino acids, with a high deuteration level that improves transverse relaxation rates in both solid and liquid samples.

## Methods

### L-amino acid oxidase stock

10 mg of LAAO powder (crude extract from the snake venom of*Cortalus admanteus*, Sigma Aldrich) were dissolved in 1 ml of 100 mM sodium phosphate, 100 mM KCl at pH 7.4. The solution can be kept at 4 °C for several weeks.

### Preparation of keto acid mix

The amino acid mix (SILEX rich growth media as powder) from Silantes was used as starting material. To obtain keto acids, 1 g of powder was dissolved in 150 ml of H_2_O. To this mixture, 10 µl of bovine liver catalase solution was added at 0 and 12 h (Sigma, fivefold water dilution from crystalline suspension, 10,000–40,000 units/mg). In total, 3–4 mg of l-amino acid oxidase (LAAO, Sigma) was used per gram powder media, added in equal amounts at 0, 3, 6, 9 and 12 h. The solution was kept shaking at 37 °C for 1 day, then lyophilized.

### Protein expression

All protein*s* were expressed in*E. coli* BL21(DE3). Two NMR model proteins were used, ubiquitin in solution, and microcrystalline chicken alpha-spectrin SH3 (SH3). In addition, the 32 kDa voltage dependent anion channel (VDAC), a beta barrel membrane protein was prepared in lipid bilayers.

For α-PET ubiquitin, a change of medium was used prior to expression. *E. coli* cells were grown in 1 L of M9 using 1 g/L of ^15^N ammonium chloride and 4 g/L of ^13^C glucose until the OD_600nm_ reached 0.6–0.8. Then cells were spun down at 7000 g at 4 °C for 20 min. The cells were re-suspended in 1L of M9 salts with 4 g of Silantes media either as received, or treated with LAAO. The cells were adapted to the new media for 30 min before induction at OD_600nm_ = 0.8 with 1 mM of isopropyl β-D-1-thiogalactopyranoside (IPTG). Ubiquitin samples, including a^13^C, ^15^N-ubiquitin reference sample were purified as previously described (Lazar et al. 1997).

Using this media exchange protocol, four different samples of ubiquitin were produced, two using ^2^H Silantes powder treated with LAAO or as received and two others using^2^H, ^13^C,^15^N Silantes powder again LAAO treated or as received.

Two samples of α-PET SH3 were produced, a media-exchanged sample (as for ubiquitin), and a second α-PET SH3 grown in the presence of glucose. Specifically, the growth was started with a low concentration of 1.25 g/L^12^C-glucose in 800 ml of M9 media. Cells were grown until OD_600nm_ reached 0.6–0.8. Then 4 g/L of treated Silantes media solubilized in 200 ml of H_2_O were added. The culture was switched to 30 °C for about 30 min until OD_600nm_ = 0.7–0.8, and protein expression was induced using 1 mM IPTG. A reference sample (^13^C,^15^N-SH3) was expressed and all samples purified as previously described (Pauli et al. 2000). In brief, the protein was purified by anion exchange chromatography (Q-TRAP, GE Healthcare) followed by gel filtration on a Superdex-75 column (GE Healthcare). The purified protein sample was extensively dialyzed against H_2_O–HCl pH 3.5 for 2 days (exchanging the dialysis solution every 12 h). The protein was then concentrated (Amicon, 3.5 kDa cut-off) to 20 mg/ml before lyophilization. The samples were resuspended in H_2_O–HCl pH 3.5 or D_2_O-HCl pH 3.5 at 15–20 mg/ml. Microcrystals were obtained using a pH shift protocol as previously described (Chevelkov et al. 2007).

α-PET VDAC was expressed at 37 °C in dilute glucose media (as for SH3) and purified and reconstituted in 2D crystalline arrays as previously described (Eddy et al. 2012; Dolder et al. 1999). The E73V, C127A, C232S variant of human VDAC was used.

### NMR measurements

Solution NMR data were recorded in a 400 MHz Bruker spectrometer at 298 K. We recorded a set of spectra to characterize the labeling pattern:^15^N-HSQC, ^13^C-HSQC in D_2_O,^1^H–^15^N TOCSY-HSQC, and 1D proton spectra. Quantification of Hα was done from a^13^C-HSQC spectrum at 950 MHz at 310 K. Transverse relaxation rates (R2) were measured at 277 K using a 600 MHz Bruker spectrometer equipped with a 5 mm cryoprobe.

The black spectrum of Fig. 6a was recorded at 105 kHz MAS on a 950 MHz Bruker spectrometer using a 0.7 mm HCND probe. All other solid state NMR data were recorded on an 800 MHz Bruker spectrometer using a 1.3 mm narrow bore HCN probe and spinning at 55 kHz MAS. We recorded cross-polarization based (H)NH, (H)CH, and (H)CANH, (H)NCAHA for resonance assignment of VDAC and SH3. We measured contacts in H(H)CH, H(H)NH spectra (SH3) and (H)C(HH)CH (VDAC) using RFDR for the proton–proton mixing. The spectra were apodized with a squared cosine function (details in Table S6). The data analysis was performed using CcpNMR and Sparky.

## Results and discussion

### Characterization of the labeling pattern

**Fig. 2:**
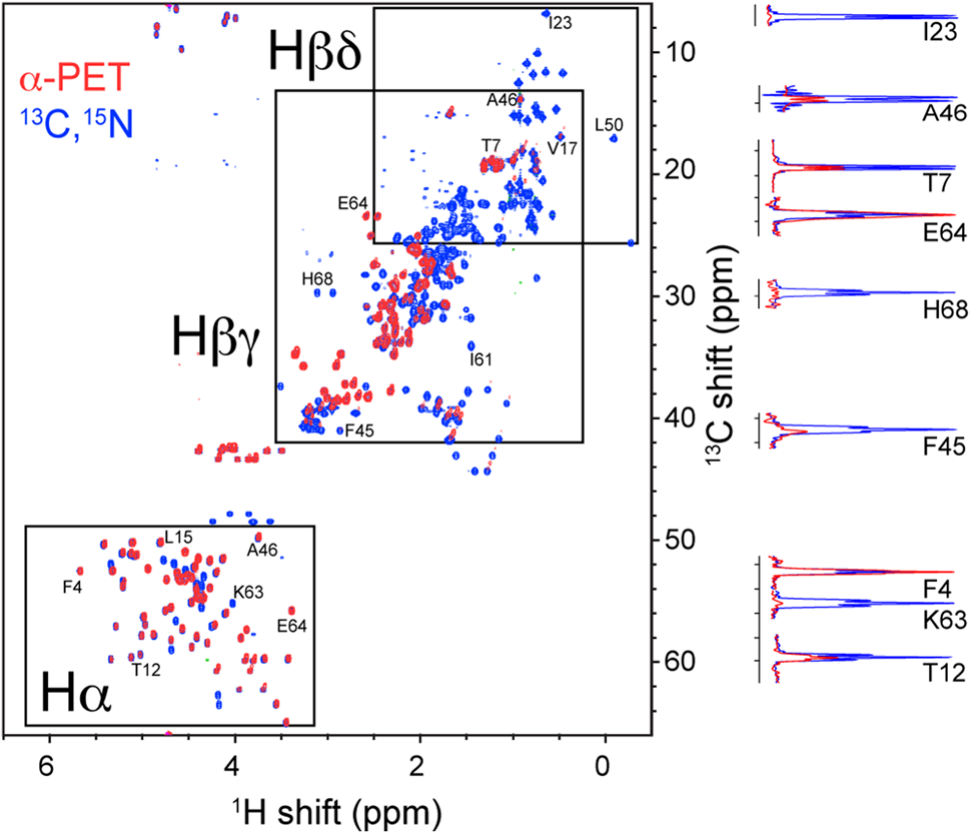
Effective incorporation of Hα protons in a ubiquitin sample, while suppressing many side-chain signals. The solution^13^C-HSQC of uniformly labelled ubiquitin (blue) is compared with α-PET ubiquitin (red). Selected slices show the intensity at backbone and sidechain sites. Intensities are not corrected for differences in T_2_

**Fig. 3:**
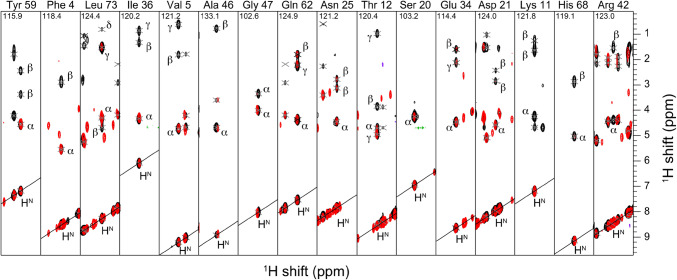
Residue-specific characterization of labeling from^1^H–^15^N TOCSY-HSQC spectra of 1 mM ubiquitin using 75 ms MLEV-17 mixing. α-PET ubiquitin (red) is compared with^15^N,^13^C-ubiquitin (black)

To measure labeling patterns on an amino acid specific basis, we recorded a ^13^C HSQC spectrum and integrated isolated peaks in the alpha region (Fig. 2). The level of Hα incorporation was determined assuming ideal incorporation of hydrophobic residues, based on complete reaction with LAAO. The uncorrected and T_2_ corrected determinations are shown in Tables S2 and S3, respectively. A ^15^N-TOCSY (Fig. 3) was recorded using a medium-range mixing time (75 ms) to assess suppression of sidechain protons. This spectrum cannot be used in a quantitative manner due to the potential for several isotopomers, differential relaxation, and relayed transfer. However, since the beta protons are relatively isolated from these effects, we could show effective suppression for most amino acid types. Figure 2 shows selected strips for each of the amino acid types of ubiquitin; the^1^H–^15^N TOCSY-HSQC of α-PET Ubiquitin (red) is compared to the ^15^N,^13^C-labeled reference sample (black). The TOCSY was implemented with MLEV-17 mixing (Bax and Davis 1985). The Hα proton was detectable for 13 of the 16 (non-proline) amino acid types present in the ubiquitin sequence. Only lysine, arginine and histidine remained deuterated at Hα. This can be explained for lysine because *Cortalus admanteus* LAAO is not able to use it as substrate (Fig S1), and the deuterated amino acids are taken up in *E. coli*, while endogenous synthesis is suppressed (Zhou et al. 1998). Although LAAO showed some activity for arginine and histidine, these two amino acids are clearly relatively poor substrates of LAAO as reported in previous studies (Arbor 1967) and also herein (Figs. S1 and S4), and therefore it appears that the resulting keto acid could not be utilized by *E. coli*, while the remaining amino acid was effectively incorporated in the protein.

Of the 13 successful amino acid types, tyrosine, phenylalanine, isoleucine, valine, alanine, threonine and aspartic acid residues show only Hα signals in the ^1^H–^15^N TOCSY-HSQC spectrum. The anabolic pathway of these residues ends with an aminotransferase reaction, with the exception of threonine, which explains the labeling. Effective aspartic acid labeling was unexpected since it enters and exits the TCA cycle, but is explained by the very high starting concentration.

The amino acid mix from Silantes (Table S1) is obtained from bacterial proteins by an HCl proteolysis and consequently glutamine, asparagine, tryptophan, and cystein are not present in the media. Therefore, glutamine and asparagine require conversion from the respective acids, which explains protonation of beta and gamma protons for these residues (Fig. 1). Glutamic acid efficiently enters and exits the TCA cycle, which may explain the incomplete suppression of beta and gamma protons.

Leucine side-chain protons were not expected, but appear to some extent due to LAAO treatment (Fig. S2). If the LAAO treatment is not performed, this sidechain labeling is not observed (Fig. S7), thus it is the crude snake venom extract that introduces leucine Hγ protons. Details of this side reaction were not investigated further, however we did follow the reaction of LAAO to test efficiency in different buffer conditions for a variety of amino acids (Figs. S1–S5).

For most amino acids, the reaction proceeded as expected, and the snake venom LAAO was particularly efficient for hydrophobic amino acids such as phenylalanine and isoleucine (Crotalus and Allen 2013; Arbor 1967). The degree of conversion to keto acids was also tested for all 20 amino acids directly in the Silantes medium. To distinguish the signal from the individual amino acid without significantly changing the composition, we used deuterated Silantes media, and added only 100 µM of each protonated amino acid. In this way, we rule out potential issues such as competitive binding to the enzyme and determine the approximate starting concentration of all amino- and keto- acids in the medium (Table 1).

**Table 1:** LAAO activity in deuterated Silantes media, as determined by solution NMR. Each amino acid was added in protonated form at a concentration of 100 µM and LAAO was added exactly as described in the methods section for expression. The remaining alpha signal intensity was used to determine the degree of conversion to keto acid

Keto acid conversion (%)	Residue	Measured Hα incorporation (%)	Residue*
90–100	Ile, Leu, Phe, Tyr, Trp, Met	90–100	Ile, Leu, Phe, Tyr, Met, Val, Ala, Gln, Asn, Thr, Ser, Glu, Asp
10–50	Val, Arg, His	10–50	
0–10	Gly, Pro, Cys, Asn, Gln, Asp, Glu, Ser, Thr, Ala, Lys	0–10	Lys, Arg, His

*Of 16 amino acids that could be quantified (see SI)

Quantification of the labeling for each residue type is tabulated in Tables S2 and S3 based on intensities extracted from^13^C-HSQC spectra. The intensities were corrected for the measured proton transverse relaxation rates (Fig. S11) and normalized based on the assumption of complete incorporation of isoleucine, phenylalanine, and leucine residues, which were cleaved completely and are known to effectively incorporate in *E. coli* (Tugarinov et al. 2003).

We also found that efficient transamination occurs when *E. coli* is grown primarily on amino acids. Some exchange still occurs at amide positions even without LAAO treatment (Figs. S6, S7), consistent with a previous report showing significant Hα labeling for TCA cycle amino acids, but only 10–50% Hα labeling for hydrophobic residues (Löhr et al. 2003).

### Glycine is labeled stereospecifically

**Fig. 4:**
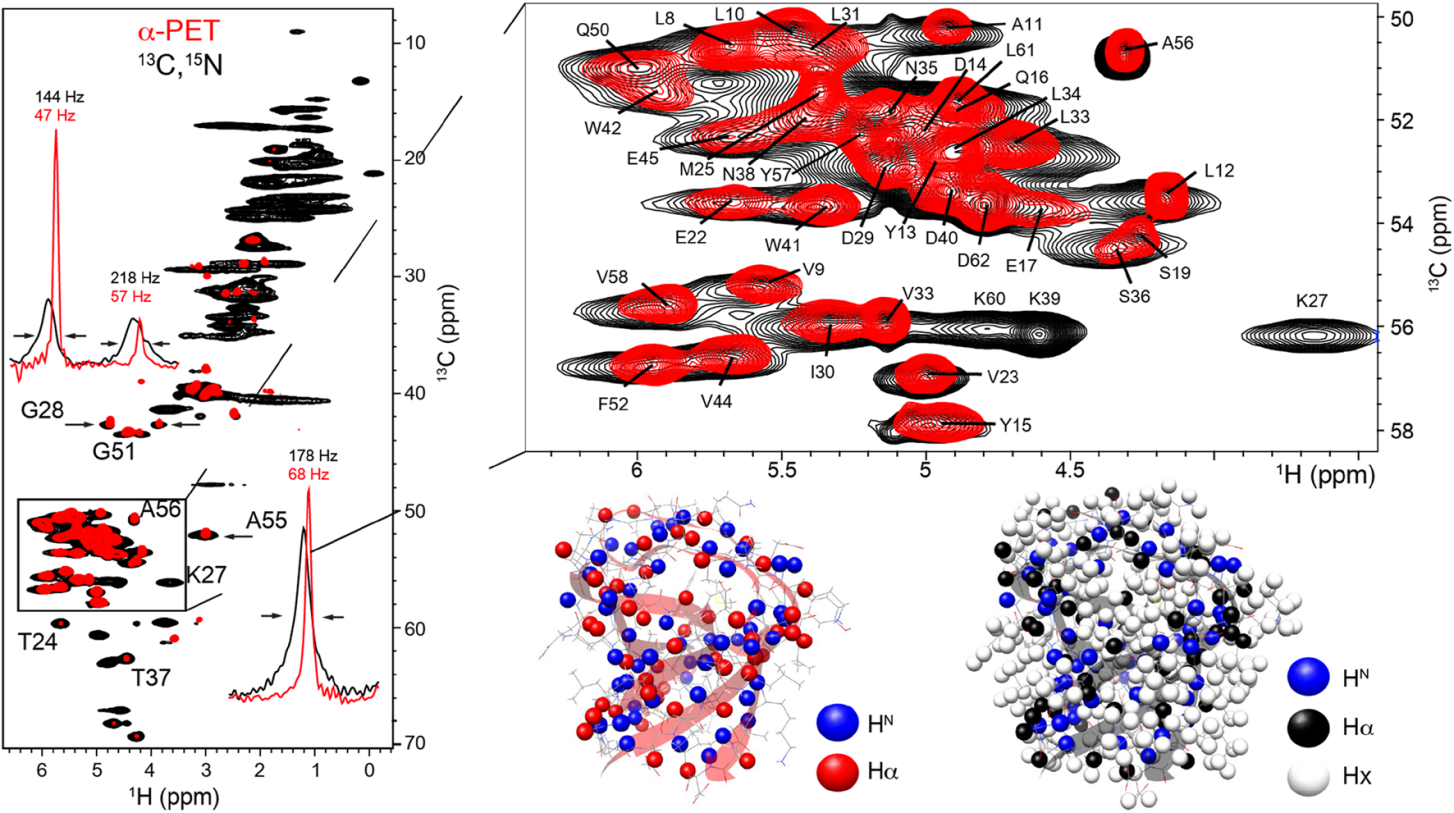
Cross-polarization based carbon-proton correlation spectra, hCH, of microcrystalline SH3 either uniformly α-PET labeled (red) and ^13^C,^15^N-labled (black) crystalized from a protonated buffer. Spectra were recorded at a magnetic field of 800 MHz and 30 °C, 55 kHz MAS. 1D slices from the spectrum indicate the improvement in linewidths for G28 (top left) and A55 (bottom right). The glycine peak intensities show stereospecific labeling with preference for R (α3 protonated) over S (α2) configuration. At the bottom right, the backbone and side-chain protons are indicated on the solution NMR structure (pdb: 1aey) for α-PET SH3 (red ribbon) and^13^C,^15^N SH3 (black ribbon)

The Hα labeling of glycine attracts particular attention, since one of the two Hα protons is labeled predominantly, resulting in stereospecific glycine labeling (Figs. 2, 4). For glycine 28, the intensity ratio between the two alpha protons for microcrystalline ^13^C,^15^N SH3 (Fig. 4, black) is 1–0.93 while the ratio is 1–0.30 for α-PET SH3 (Fig. 4, red). This effect was observed for glycine in all the samples tested, based on signal intensity in HSQC and CP-HSQC spectra. We also observed a considerable reduction in line width, by more than a factor of three.

Glycine can be produced from serine by hydroxymethyltransferase, from threonine by l-allo-threonine aldolase, or through serine–glyoxylate or alanine–glyoxylate transaminases. Information in *E. coli* is limited, but analysis of other organisms using tritiated water indicates that the stereo specificity depends on the pathways involved (Yoshimura et al. 1996; Dunathan et al. 1968; Wellner 1970). If serine transhydroxymethylase acts in tritiated water, the resulting glycine will predominantly be the S configuration, but if liver transaminase acts then R will be the predominant configuration. Note that in our case, each enzyme will produce the reverse stereoisomer because the starting amino acid is deuterated, and the enzymatic reaction occurs in protonated water. By examination of NOE spectra of ubiquitin we observed a cross peak between the glycine 47 Hα and isoleucine 45 H^N^, which according to the known structure, indicates that glycine was predominantly the R configuration. This is consistent with the stereospecific labelling approach reported previously using cell free extracts (Loscha and Otting 2013), but results in the opposite labeling, since we expressed in H_2_O rather than D_2_O. We can therefore rule out deuterated glycine from the medium as the main source of stereospecific glyine found in the expressed protein.

### Resolution and structural data under MAS conditions

To demonstrate that the α-PET labeling scheme results in improved resolution, we prepared a microcrystalline sample of α-spectrin SH3 according to established crystallization protocols (Pauli et al. 2000). The Hα line width is significantly reduced for α-PET SH3 and the effect is particularly improved for certain residues, by a factor of two and above (Fig. 4). The proton resolution is also superior to labeling with deuterated glucose in otherwise protonated media (Medeiros-Silva et al. 2016) (Fig. S8). To characterize the narrowing of the homogeneous part of the lines, the bulk T_2_´ relaxation times at 55 kHz was measured for Hα, Cα, and CO from 1D (HCAN)H, (HCON)H and (HCA)HA spectra by integrating the full signal. The Hα T_2_´ of 3–4 ms for α-PET SH3 is a dramatic improvement compared to 1 ms for the fully protonated sample (Fig S9).

The Hα T_2_´ of α-PET SH3 crystallized in 100% D_2_O buffer ranged from 7 to 15 ms, an improvement over the amide protonated sample large enough that we can directly observe an increase in resolution in the 1D spectrum (Fig S9). The improvement is further characterized for select residues in Figure S12. The H^N^ signals were almost completely removed in the D_2_O buffer.

Sequential resonance assignment in Fig. 4 were made using a (H)NCAHA spectrum, and are consistent with those previously reported (Xue et al. 2017). SH3 has 62 residues, of which, two are prolines and the N-terminal seven residues and residues 46–48 are flexible and are therefore not observed using cross-polarization based transfer experiments. Thus 50 Hα peaks are expected for^13^C,^15^N SH3. For α-PET SH3 lysine, arginine and histidine are not expected. Thus only 41 Hα peaks are expected and indeed 41 peaks were readily identified in (H)NCAHA spectra.

**Fig. 5:**
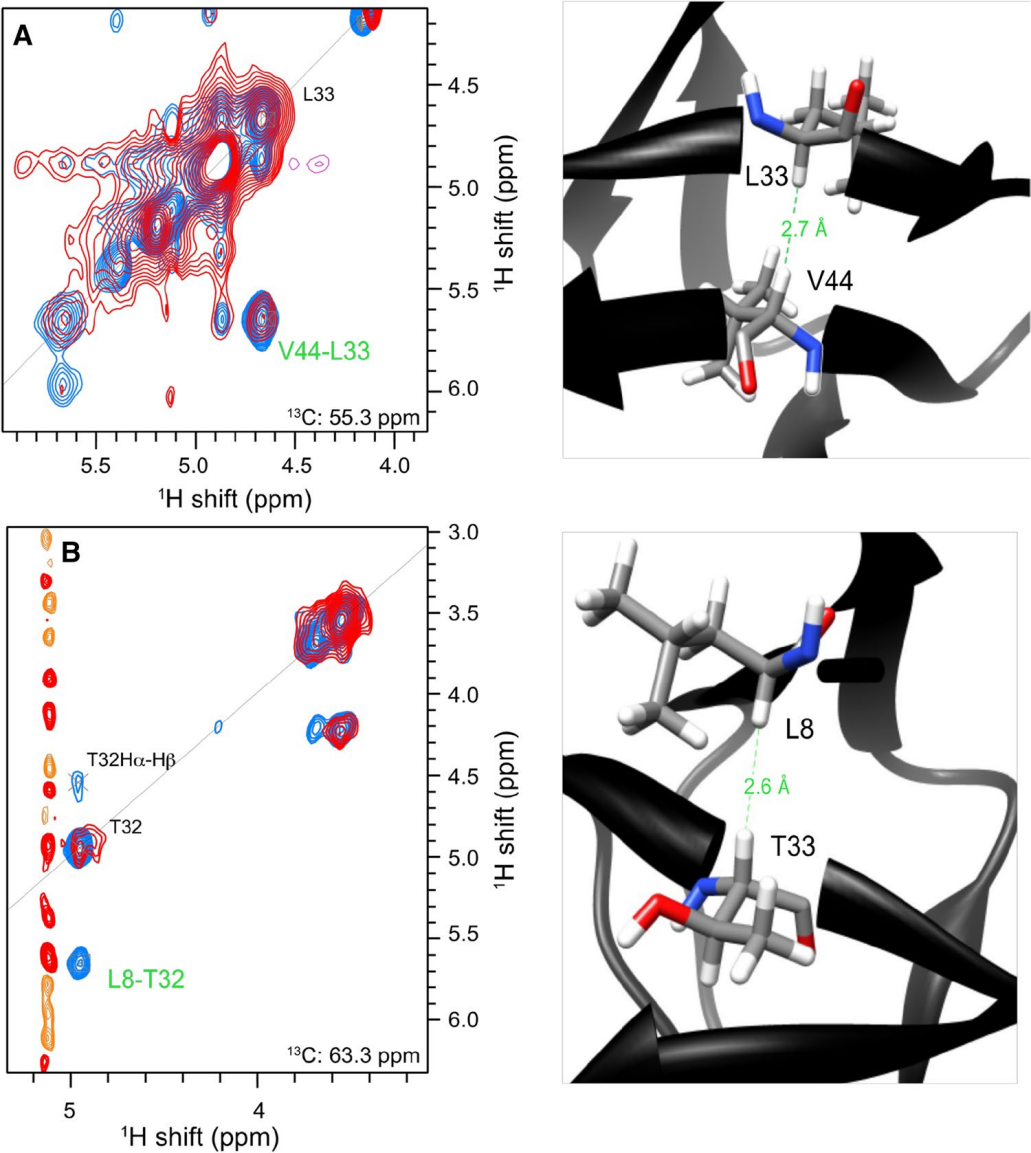
Long-range distance information is highlighted in a 3D H(H)CH spectrum of α-PET SH3 (pdb: 1aey) in D_2_O (blue) and in H_2_O (red). **a** Shows a contact between L33 Hα and V44 Hα. In**b**, the contact between T32 Hα and L8 Hα is readily observed in D_2_O (in blue) while it is much weaker in the presence of additional protons in H_2_O (in red). Recorded in a 800 MHz Bruker spectrometer at 30 °C and 55 kHz MAS

Figure 5 shows a comparison between α-PET SH3 in fully protonated buffer (red) and α-PET SH3 in fully deuterated buffer (blue) in which long-range structural restraints were measured. To characterize the benefit of the restraints present with α-PET labeling, we manually selected peaks in the H(H)CH and H(H)NH spectra, and used automated shift matching (0.05, 0.5 and 0.5 ppm tolerance, in^1^H, ^13^C and^15^N, respectively) to identify contacts. Of 114 automatically assigned peaks from the 3D H(H)NH of α-PET SH3 in protonated buffer, two unambiguous contacts were identified, of which one is a long-range H^N–^H^N^ contact. For the 3D H(H)CH, 132 peaks were selected, and seven unambiguous contacts were identified, five of which are long-range restraints. However, for the H(H)CH spectrum in deuterated buffer we found 150 contacts, of which eight are unambiguous restraints, seven of which were long range corresponding to either H^N^–Hα or Hα–Hα. One of the additional contacts identified in fully deuterated buffer is highlighted in Fig. 5b. This method clearly improves the number of structural restraints available at 55 kHz MAS, and in particular, the unambiguous restraints, a metric that is crucial for the convergence of commonly used structure calculation methods. A concern with Hα detection is the presence of water and other solvent signals in this spectral region. Therefore good water suppression is needed, but as demonstrated here for samples in both H_2_O and D_2_O, control of the water is possible even without gradient methods.

For resonance assignment, the α-PET labeling approach benefits from the implementation of new proton detected NMR pulse sequences focused on Hα detection that were recently developed for > 100 kHz MAS (Stanek et al. 2016). So far, proton detected MAS NMR structures were mostly based on H^N^ detected experiments or more recently on fully protonated samples that are best investigated using >100 kHz MAS. (Cala-De Paepe et al. 2017) New possibilities are opened with the α-PET approach, allowing effective structural measurements with the inherently more sensitive equipment for ~ 60 kHz spinning.

The method was also successful for a more challenging system, the human voltage-dependant anion channel (VDAC). The lipid bilayer structure of this protein has been investigated through MAS NMR spectra of VDAC in liposomes (Schneider et al. 2010) and in 2D crystalline arrays (Eddy et al. 2015), and narrow proton resonances were reported for a perdeuterated sample.(Eddy et al. 2015a, b) With α-PET labeling, we also observed narrow amide proton linewidths of 150 Hz in H_2_O, while ~ 100 Hz lines were observed using D_2_O buffer, which is slightly better than the ~ 120 Hz linewidths observed for perdeuterated and H^N^ back-exchanged protein. This indicates that the non-exchangeable protons are slightly narrower, and that Hα labeling does not significantly impact the spectral quality. In this D_2_O-exchanged buffer, less improvement in Hα T_2_´ (Fig. S9) was observed as compared with the SH3 domain, which is not unexpected, since approximately half the amide protons were protected from exchange (Fig. S9B).

**Fig. 6:**
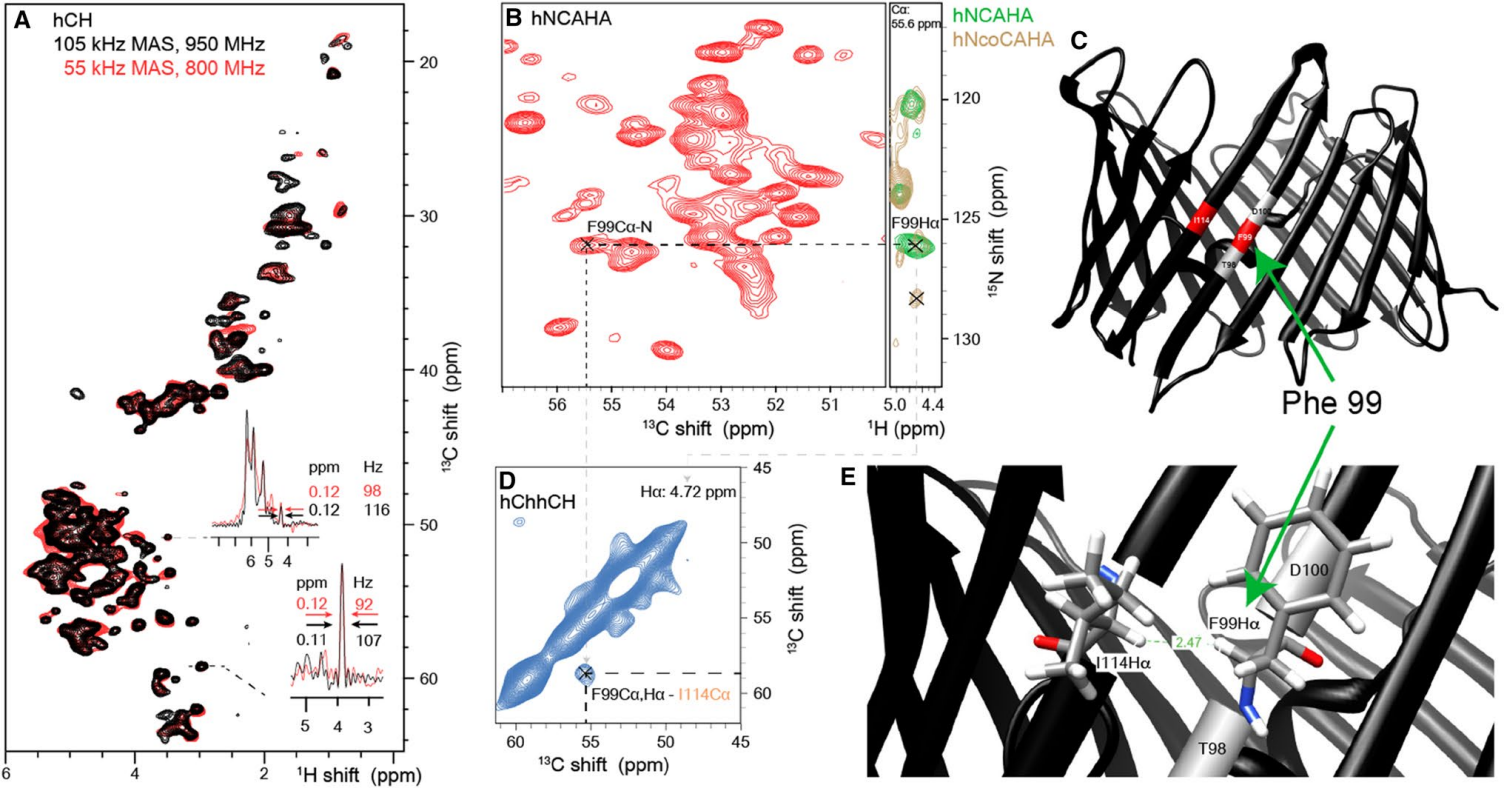
Identification of a cross beta strand contact (F99–I114 Hα) in the beta barrel membrane protein VDAC in lipid bilayers. **a** Shows, the comparison of the (H)CH spectrum at 105 kHz on a 950 MHz spectrometer (black) and at 55 kHz on an 800 MHz spectrometer (red). **b** Shows a^13^C–^15^N projection of a (H)NCAHA spectrum. F99 Hα is assigned from the strip comparing (H)NCAHA (green) and (H)N(CO)CAHA (brown). In**c** and **e**, the contact is shown on the X-ray structure of mouse VDAC (pdb: 2jk4). **d** Shows the F99–I114 cross-peak in the carbon–carbon 2D plane of the (H)C(HH)CH spectrum at the proton frequency of F99, 4.72 ppm

To further characterize the potential spectral resolution, α-PET VDAC was measured at 105 kHz MAS at a 950 MHz spectrometer (black in Fig. 6a). Surprisingly, the same line width was obtained at 110 kHz MAS at 950 MHz (~ 110 Hz) and at 55 kHz MAS at 800 MHz (~ 95 Hz), showing that the inhomogeneous contributions are dominating the linewidth at 55 kHz. This shows that even for a highly homogeneous preparation of a membrane protein, α-PET labeling efficiently reduces the proton dipolar broadening at 55 kHz.

The protection from solvent exchange observed for VDAC highlights an issue with perdeuteration for proteins that lack refolding protocols. Perdeuteration of membrane proteins (Medeiros-Silva et al. 2016; Ward et al. 2011) and large complexes (Andreas et al. 2016) in *E. coli* often results in deuterated amides that cannot be exchanged with protons from water. Such exchange protected regions of the protein become inaccessible in the perdeuteration and back-exchange approach, limiting the analysis to solvent accessible regions (Andreas et al. 2015; Chevelkov et al. 2006; Fricke et al. 2017; Zhou et al. 2007a, b; Ward et al. 2015), although such limited exchange phenomena can also be used to obtain functional information (Ward et al. 2011; Böckmann and Guittet 1997; Agarwal et al. 2010). Using α-PET labeling, we are now able to detect both exchangeable as well as non-exchangeable amide protons in highly deuterated samples as shown previously for amino acid based media (Löhr et al. 2003).

Due to the size of the protein, unambiguous assignment of important cross-strand contacts was not possible in a 3D H(H)CH spectrum of VDAC. We therefore applied the better resolved 3D (H)C(HH)CH spectrum to measure cross-strand contacts (Fig. 6). VDAC assembles as a beta barrel, a topology that places cross-strand Hα pairs in close proximity (~ 2.3 Å), and much closer than sequential Hα spins, which are separated by about 4.5 Å. 28 Hα–Hα contacts were detected from this spectrum, of which we show the cross strand contact between residue phenylalanine 99 and isoleucine 114, which was assigned based on the existing^13^C and ^15^N assignments of this protein (Eddy et al. 2015a, b) and (H)NCAHA and (H)N(CO)CAHA spectra. The current published assignments (32% of 283 residues) of VDAC do not allow a characterization of all 28 peaks. However, resolving 28 peaks is significant, considering that only ~ 4 amide–amide or alpha–alpha contacts are available in each transmembrane beta sheet interface, of which VDAC has 19. Further analysis of the expected contacts in VDAC is show in in Fig. S10 This demonstrates a successful implementation of α-PET labelling for structure determination in a challenging 32 kDa membrane protein embedded in lipid bilayers, where structural restraints are particularly difficult to identify (Eddy et al. 2015).

### α-PET labeling for solution NMR

**Fig. 7:**
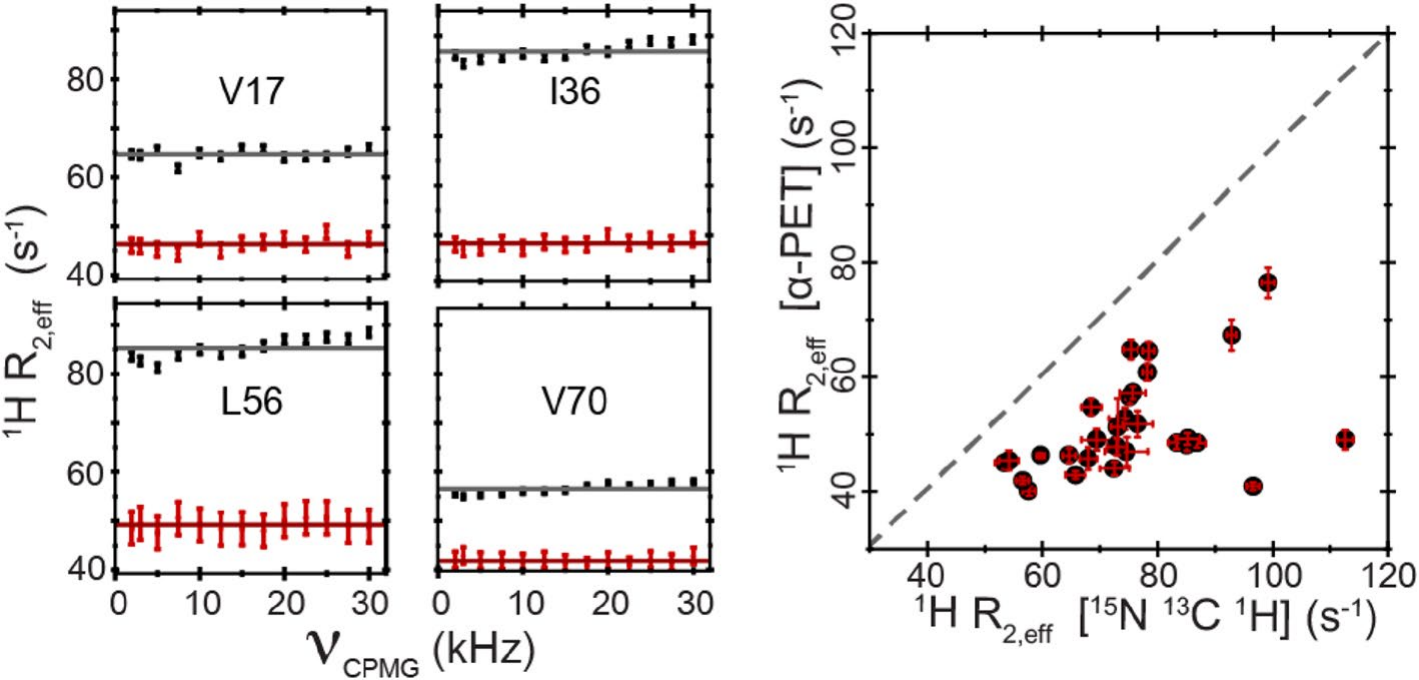
Selected residues showing the reduction in proton (Hα) R_2_ relaxation rates with α-PET labeling (red) as compared with full protonation (black). The correlation plot (right) shows a reduction for all residues. The data is from ubiquitin samples exchanged in 100% D_2_O at 277 K and measured at a 600 MHz spectrometer

The α-PET labeling approach is also beneficial for the study of proteins in solution, when deuteration is needed to reduce transverse relaxation rates (LeMaster and Richards 1988; Torchia et al. 1988). Figure 7 and Tables S4–S5 show the reduction in R_2_ relaxation rates due to the high level of deuteration in α-PET labeled Ubiquitin. Such improvement in relaxation rates is important for the study of protein dynamics. For example in detection of Hα relaxation dispersion, fractional deuteration was used to improve R_2_ (Lundström et al. 2009; Vallurupalli et al. 2009). The current labeling incorporates the alpha positions at 100% for most residues, with a high overall deuteration level, which improves sensitivity as compared with random fractional deuteration.

## Conclusions

Here, we introduced a new method to label Hα protons in a protein without significant isotopic scrambling, and demonstrated how this new sensitive magnetic probe in the backbone of the protein adds new structural information even at below 60 kHz MAS. The α-PET labeling approach has several advantages, (i) adaptation of the cells to D_2_O is not required, (ii) it gives similar yields as deuterated expression in M9 media, and (iii) costs are similar to production of deuterated proteins. It is expected to be particularly useful for deuteration of proteins that lack refolding protocols, such as membrane proteins.

In this demonstration, we used a commercially available crude snake venom extract to generate keto acids. This approach results in the designed incorporation of alpha protons for Tyr, Phe, Leu, Ile, Gly, Gln, Asn, Asp, Glu and Met. In the future, further optimization of the method might entail other LAAOs with different substrate specificity, perhaps in combination with auxotrophic strains to limit unwanted reaction pathways. In addition, other amino acid mixtures or expression systems could be investigated. This might allow labeling of lysine, arginine, and histidine, which were currently left deuterated.

## Supporting Information

Quantification of labeling patterns, LAAO activity, measurement of relaxation times under 55 kHz MAS, Hα R_2_ in solution and spectral acquisition parameters.

## Electronic supplementary material

Below is the link to the electronic supplementary material.

Supplementary material 1 (PDF 3597 KB)
