# Supplementary material for: Alpha protons as NMR probes in deuterated proteins
Source: J Biomol NMR. 2019 Feb 14;73(1):81–91. doi: 10.1007/s10858-019-00230-y (PMC6441447; doi:10.1007/s10858-019-00230-y)
Supplement: Supplementary file 1 — Supplementary material 1 (PDF 3597 KB) [file 10858_2019_230_MOESM1_ESM.pdf]

Supporting information to:

Alpha protons as structural NMR probes in deuterated proteins

Kumar Tekwani Movellan[1], Eszter Najbauer[1], Supriya Pratihar[1], Michele Salvi[1], Karin Giller[1], Stefan Becker[1], Loren B. Andreas[1]

1. Department of NMR based Structural Biology, Max Planck Institute for Biophysical Chemistry, Am Fassberg 11, Göttingen, Germany

Amino acid composition before treatment	
**Amino acid**	**mole percent**
Asp	20.6
Thr	3.7
Ser	4.1
Glu	11.1
Gly	11.1
Ala	14.8
Val	3.0
Met	1.8
Ile	2.1
Leu	5.3
Tyr	1.8
Phe	2.4
His	9.7
Lys	3.4
Arg	2.3
Pro	3.0

Table S1: Amino acid composition of Silantes media as supplied by the manufacturer, with negligible concentrations of carbohydrates (less than 30 mg per liter).

Residue	Number of residues	Reference sample	αPET sample	ratio	stdv	percentage
Tyr	1	2.18	3.16	1.451	*	145.1
Phe	2	2.24	2.34	1.046	0.04	104.6
Leu	8	3.57	3.63	1.015	0.11	101.5
Ile	7	2.79	2.64	0.944	0.08	94.4
Val	4	3.49	2.41	0.692	0.07	69.2
Ala	2	3.19	2.05	0.644	0.02	64.4
GlyHα3	3	2.53	3.24	1.280	0.07	128.0
GlyHα2	3	4.41	1.05	0.239	0.01	23.9
Gln	4	2.91	2.79	0.960	0.07	96.0
Asn	1	3.22	3.82	1.186	*	118.6
Thr	7	3.09	1.05	0.341	0.01	34.1
Ser	2	2.67	1.27	0.476	0.01	47.6
Glu	5	2.77	2.87	1.038	0.03	103.8
Asp	2	3.08	3.53	1.146	0.02	114.6
Lys	5	3.56	-	0.000	*	0.0
Arg	4	4.42	-	0.000	*	0.0
His	1	2.95	-	0.000	*	0.0
Met	1	4.76	4.52	0.950	*	95.0
	62					

Table S2: Incorporation level at the Hα position by amino acid type as estimated from ^{13}C-HSQC spectra without correcting for differences in T_2 relaxation. Spectra were recorded on a 950 MHz Bruker spectrometer using 30 ms indirect evolution for both α-PET and U-^{13}C,^{15}N-labeled samples in D2O. Water suppression by saturation or selective pulsing was not used, since the alpha protons overlap with water. The data was processed using a sine squared function in both dimensions and using 8k indirect points. Peak intensities where used to estimate labelling efficiency. Amino acids that do not scramble, and for which LAAO was effective (Ile, Phe, Leu) were used to normalize the spectra. The largest source of error is not due to the signal to noise ratio, and we therefore expect that it comes from differences in relaxation between the deuterated and protonated samples. We therefore corrected for T_2 in Table S3.

Residue	Number of residues	Reference sample	αPET sample	ratio	stdv	percentage
Tyr	1	1.20	1.25	1.040	*	104.0
Phe	2	1.32	1.29	0.984	0.03	98.4
Leu	8	1.23	1.25	1.017	0.02	101.7
Ile	6	1.28	1.28	1.001	0.03	100.1
Val	3	1.22	1.26	1.039	0.04	103.9
Ala	2	1.21	1.27	1.046	0.01	104.6
GlyHaa	0	*	*	*	*	*
GlyHab	0	*	*	*	*	*
Gln	4	1.25	1.31	1.048	0.01	104.8
Asn	1	1.22	1.31	1.075	*	107.5
Thr	3	1.35	1.36	1.007	0.09	100.7
Ser	1	1.24	1.29	1.038	*	103.8
Glu	4	1.27	1.31	1.027	0.03	102.7
Asp	1	1.19	1.29	1.078	*	107.8
Lys	5	1.24	*	*	*	*
Arg	4	1.17	*	*	*	*
His	1	1.24	*	*	*	*
Met	1	1.20	1.26	1.044	*	104.4
	47					

Table S3: Incorporation level at the Hα position by amino acid type as estimated from ^{13}C-HSQC spectra corrected by the Hα T_2. The intensities from Table S2 were corrected by the T_2 measured at the Hα position of each individual amino acid. Peak intensities were used to estimate labelling efficiency. Amino acids that do not scramble, and for which LAAO was effective (Ile, Phe, Leu) were again used to normalize the spectra. With T_2 correction, Val, Ala, Ser and Thr show higher incorporation, of about 100%. The correction of the T_2 could only be made reliable for isolated peaks that do not overlap with other peaks or with water (only 47 peaks have been used in this case).

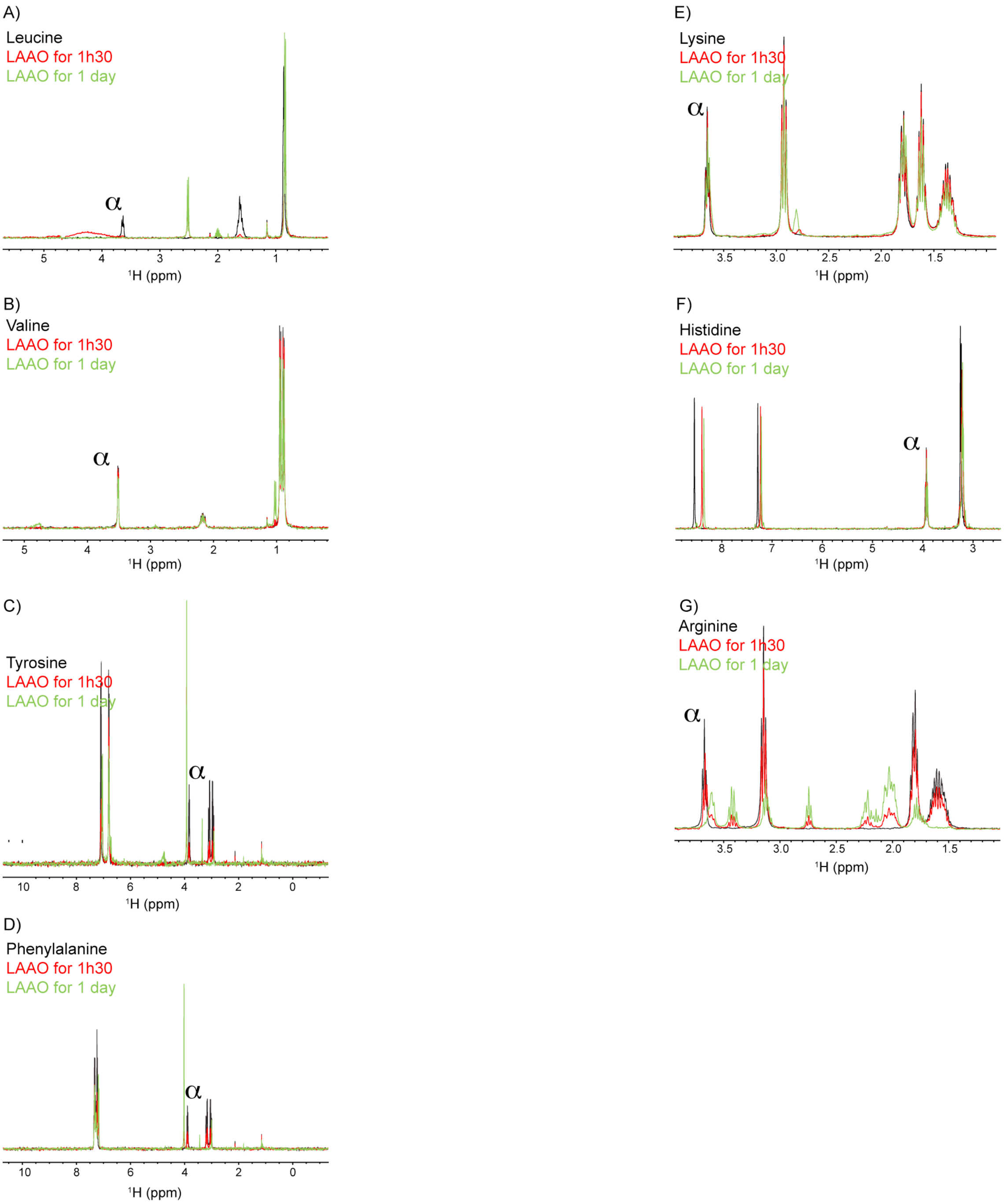

Figure S1: LAAO activity for different amino acids in solution. Conversion was effective for leucine (A), tyrosine (C), phenylalanine (D) and arginine (G), and not effective for valine (B), lysine (E) and histidine (F). Some activity was observed for histidine at lower concentration (see Figure S4).

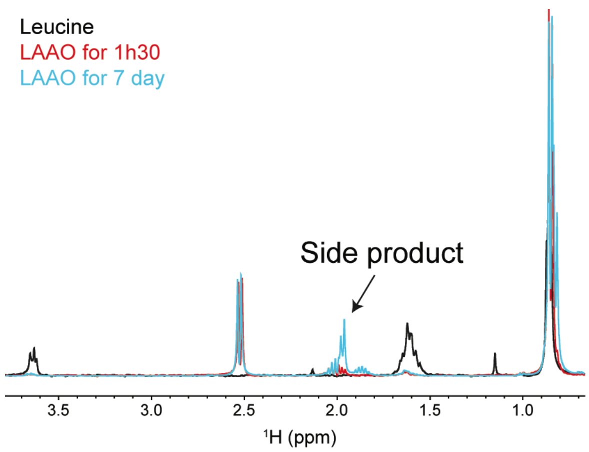

Figure S2: Side products from LAAO treatment on Leucine

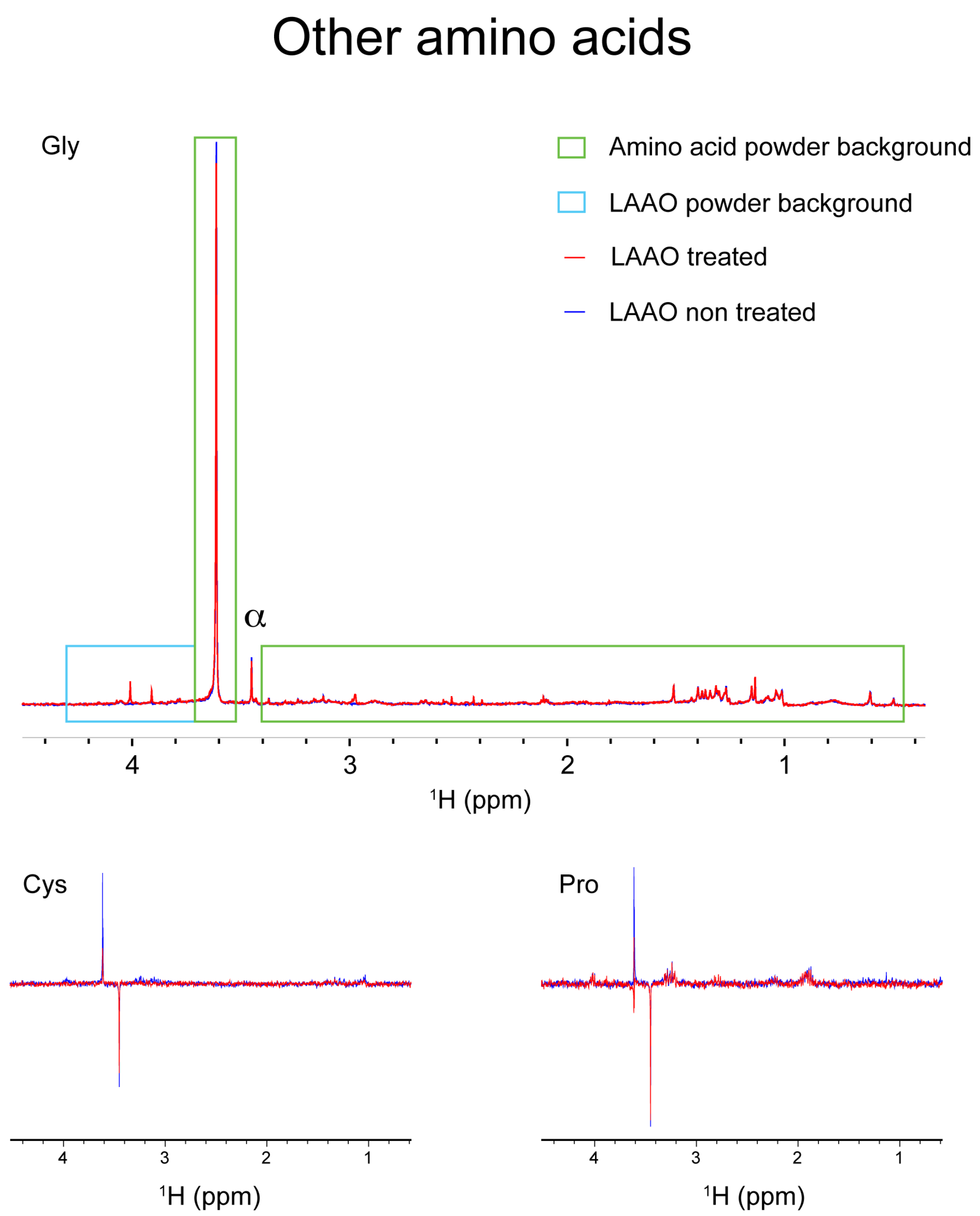

Figure S3: LAAO treatment efficiency on a deuterated amino acid powder for different amino acids. 50 uM of the respective amino acid were dissolved in deuterated powder. The Glycine spectrum was used to cancel the noise from the powder (green box in the glycine spectrum) and the noise from the LAAO (bleu box in the glycine spectrum) by doing the difference with the respective spectrum (Glycine Hα appears as a negative peak). Gly, Cys and Pro were not affected by the LAAO treatment.

Polar/Charged amino acids

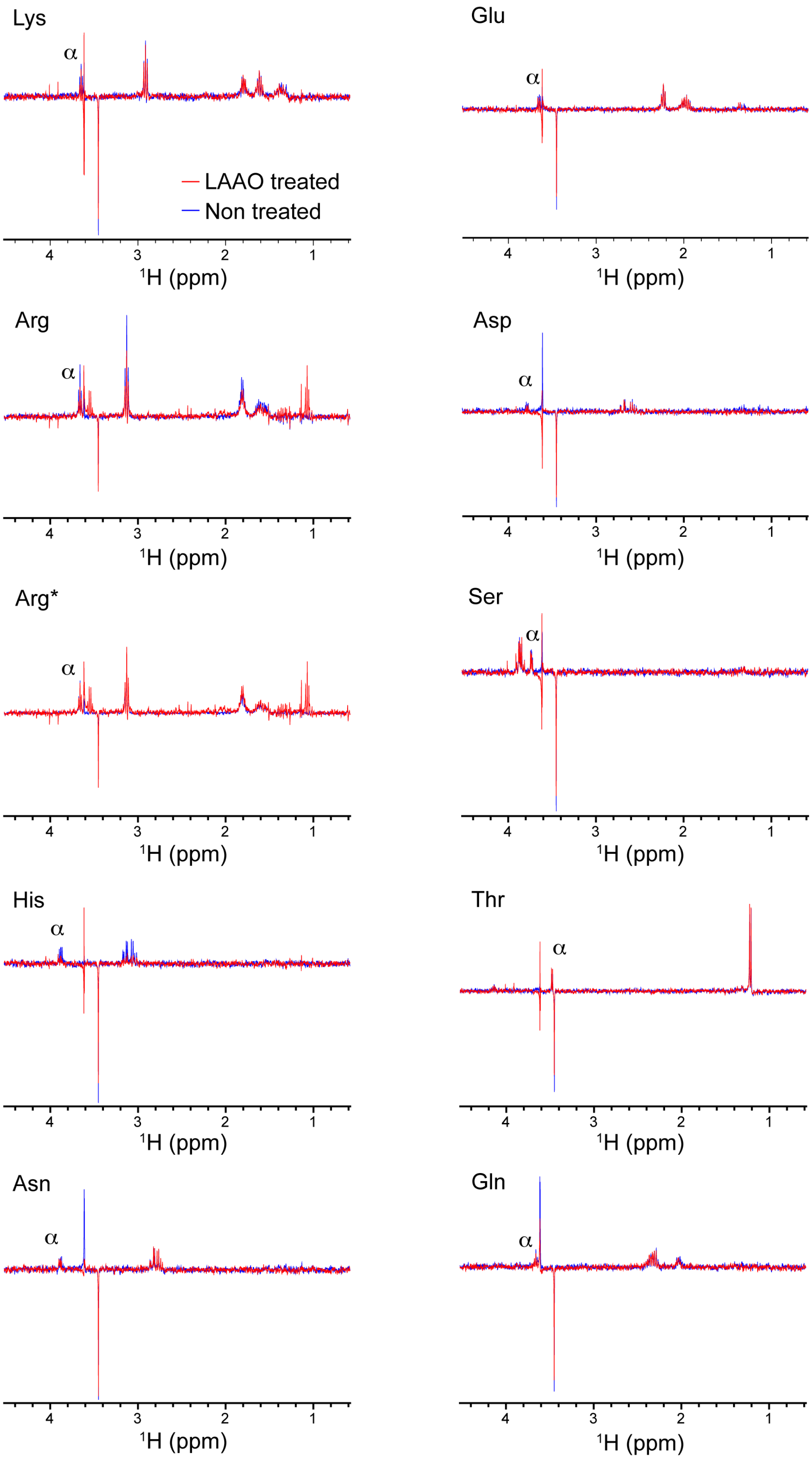

Figure S4: LAAO treatment efficiency on a deuterated amino acid powder for polar/charged amino acids. 50 uM of the respective amino acid were dissolved in deuterated powder. The Glycine spectrum was used to cancel the background by taking the difference (Glycine Hα appears as a negative peak). Arg* corresponds to the Arg spectrum scaled as a function of the triplet (CH2), since the arginine concentration was different in the reference and LAAO treated samples. The LAAO enzyme is active on His and Arg, but results in only partial production of the keto acid. For all the other polar and charged amino acids Lys, Glu, Asp, Asn, Gln, Ser and Thr LAAO has no effect.

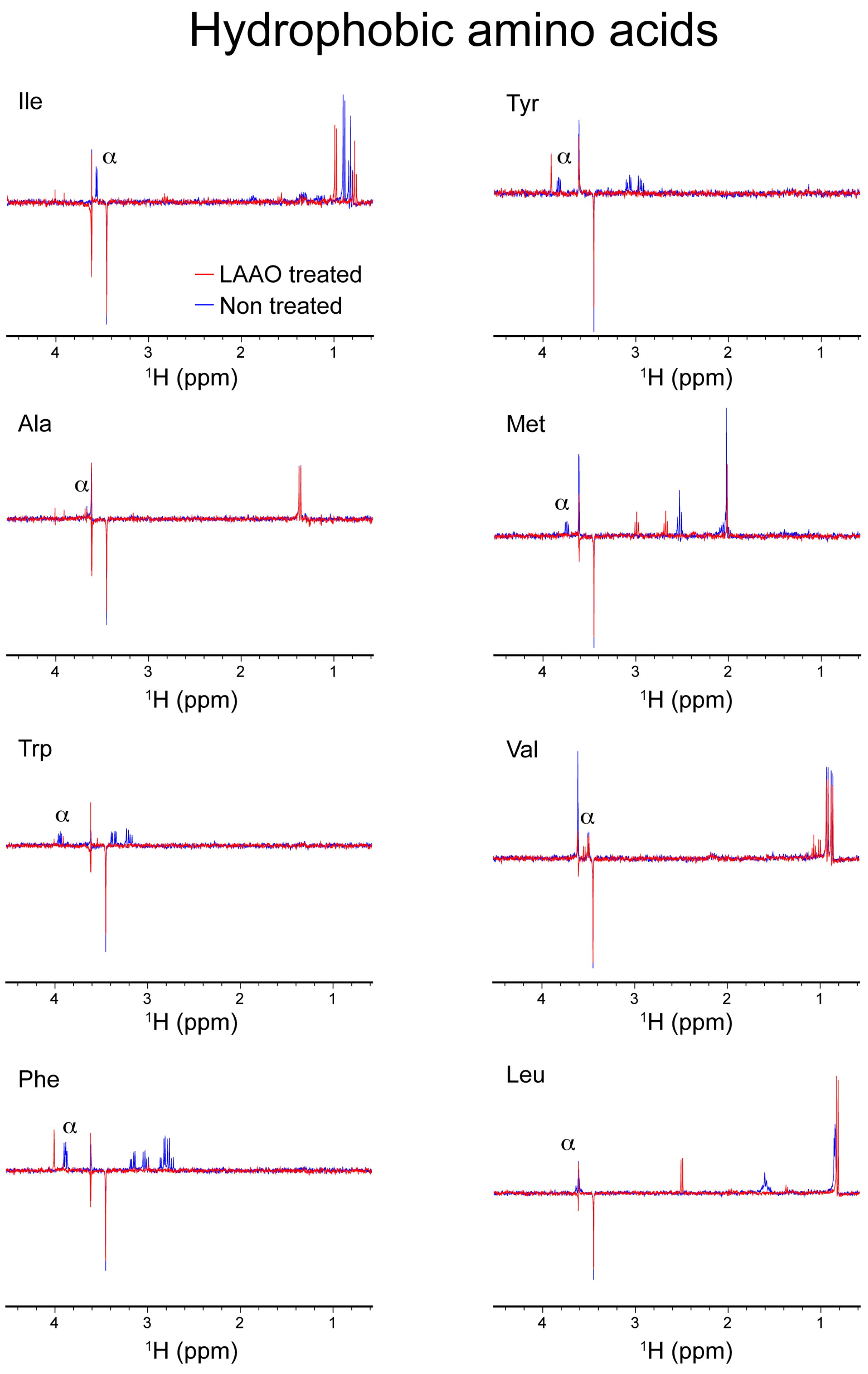

Figure S5: LAAO treatment efficiency on a deuterated amino acid powder for hydrophobic amino acids. 50 uM of the respective amino acid were dissolved in deuterated powder. The Glycine spectrum was used to remove the background signal by taking the difference spectrum (Hα of Glycine appears as a negative peak). LAAO treatment is 100% effective for Ile, Leu, Phe, Trp, Tyr and Met. The LAAO treatment has some activity on Val, however, is not active for Ala in the deuterated amino acid media.

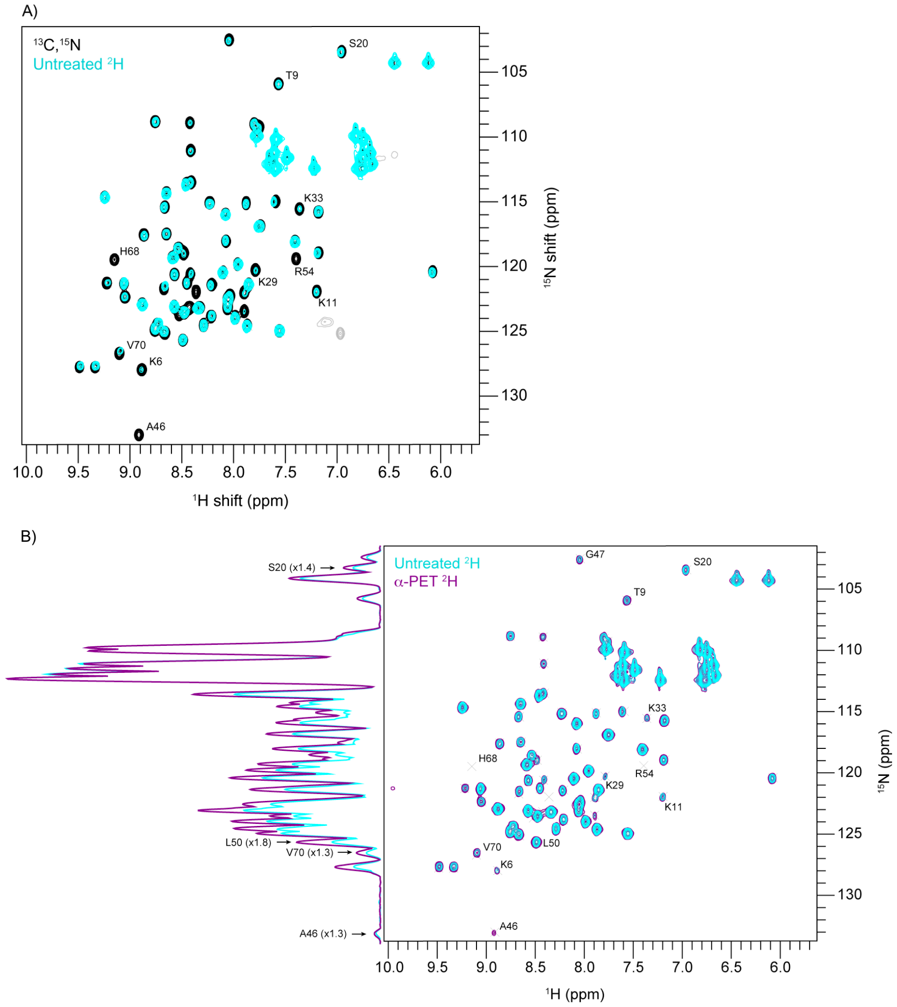

Figure S6: Transaminase and other enzyme activity in *E. coli* is sufficient to exchange the amide position of many residues, with and without LAAO treatment. ^{2}H Silantes (without ^{15}N labelling) was added to the culture after growth in ^{15}N M9 medium. Peaks that remain are labelled through amino acid synthesis pathways or through transaminase. Suppression of sidechain protons (Fig. S8-S9) indicate that synthesis pathways are not a significant contribution. In A, ^{13}C,^{15}N-ubiquitin (black) and untreated ubiquitin (cyan) shows the inherent transaminase activity *E. coli*. In B, ^{15}N-HSQCs of LAAO treated (purple) and untreated (cyan) ubiquitin (starting with ^{2}H Silantes) shows that treatment helps to introduce a higher level of amide signal for many amino acids.

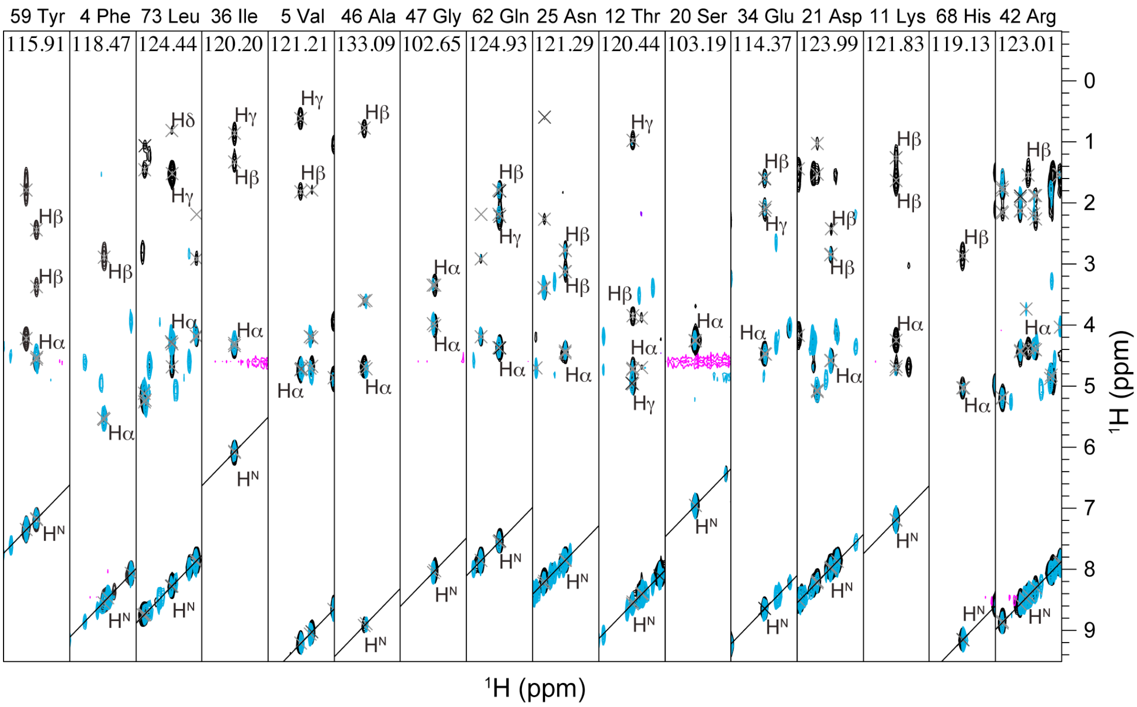

Figure S7: Amino acid labelling pattern of α-PET Ubiquitin without LAAO treatment. The ^{15}N-TOCSY of $^{15}N,^{13}C$-Ubiquitin (black) is compared with untreated α-PET-Ubiquitin (cyan).

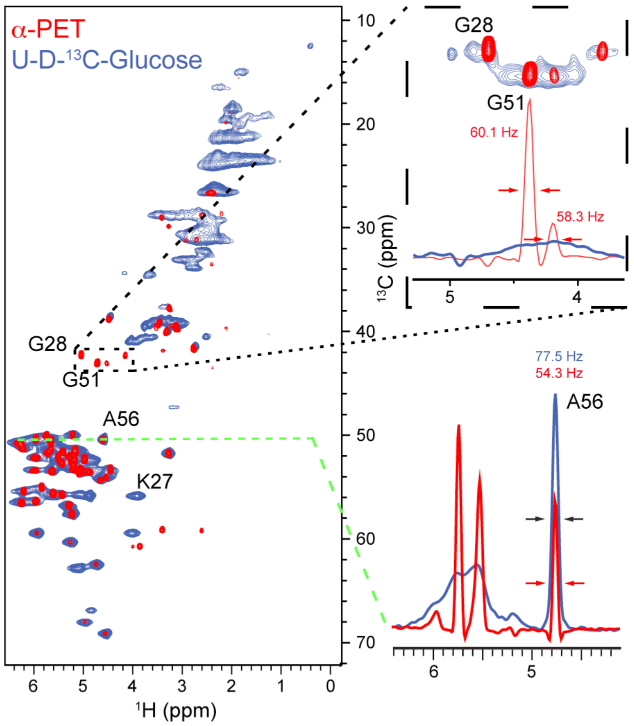

Figure S8: Comparison of spectral quality when labeling with deuterated glucose in otherwise protonated media. Both spectra were recorded at a MAS spinning frequency of 55 kHz on a Bruker 800 MHz spectrometer. The improvement in resolution with α-PET labeling is compared for selected regions of the 2D spectrum. This labeling scheme has been dubbed inverse fractional deuteration (iFD)[1].

A)

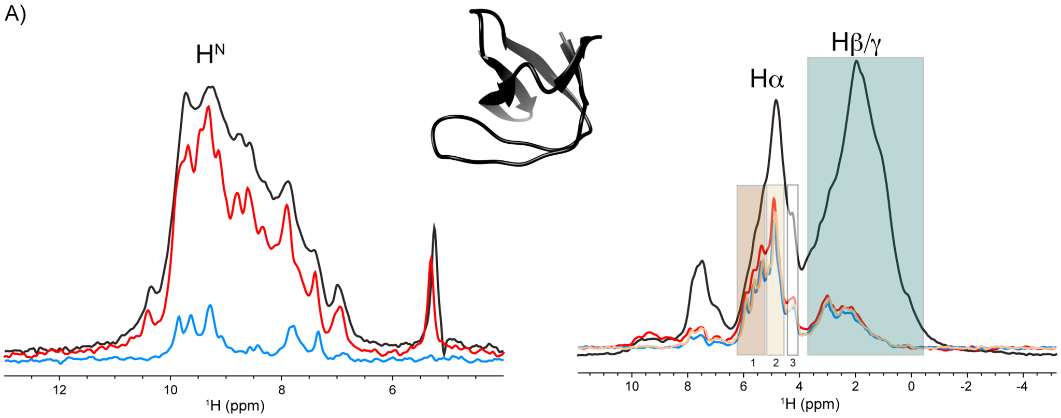

T2' (ms)	H^N	$H\alpha_1$	$H\alpha_2$	$H\alpha_3$	Hβ/γ
Full protonation	1.245		1.39		0.878
α-PET in H2O	3.58	2.79	3.1	5.64	2.36
α-PET in D2O	*	7.04	8.59	15.22	4.47
α-PET in D2O*	*	7.59	8.49	16.2	4.17

B)

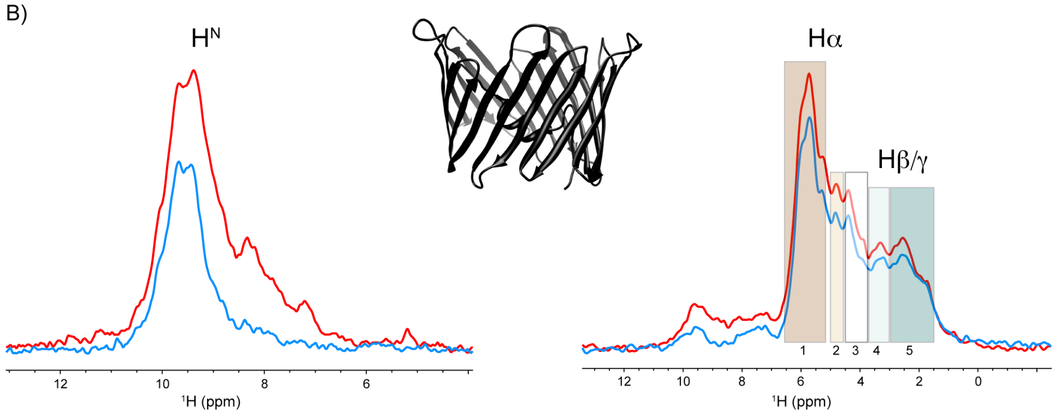

T2' (ms)	H^N	$H\alpha_1$	$H\alpha_2$	$H\alpha_3$	$H\beta/\gamma_4$	$H\beta/\gamma_5$
α-PET in H2O	2.72	1.85	2.84	2.67	2.25	1.96
α-PET in D2O	2.87	2.36	3.97	3.83	2.56	2.04

Figure S9: Transverse relaxation (T2') is compared for α-PET samples in 100% deuterated buffer and 100% protonated buffer. Data were recorded at 800 MHz, with 55 kHz MAS and filtered through cross polarization based hNH or hCH spectra for H^N or H^C relaxation times, respectively. In A, H^N and H^C relaxation times for the microcrystalline α-spectrin SH3 domain labelled with α-PET in protonated buffer (red), α-PET in deutereted buffer (blue), α-PET using the media exchange protocol in deutereted buffer (brown), and ^{13}C,^{15}N in protonated buffer (black). The first point of the decay curves are shown above the table in the corresponding colors. In B, the bulk T_2' relaxation times of the α-PET labelled 32kDa membrane protein VDAC are tabulated. Again, the first point in the decay curves are shown in the corresponding colors.

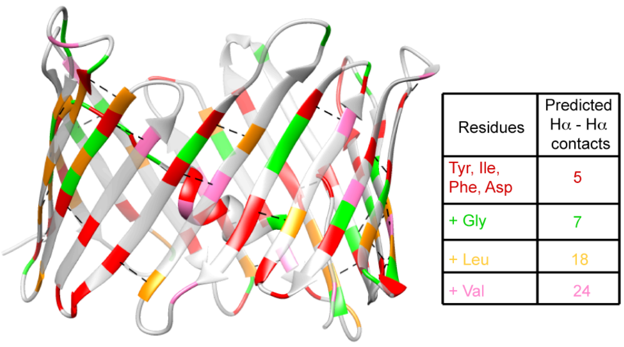

Residues	Predicted Hα - Hα contacts
Tyr, Ile, Phe, Asp	5
+ Gly	7
+ Leu	18
+ Val	24

Figure S10: Predicted Hα– Hα contacts accordingly to α-PET labeling mapped into the VDAC NMR structure. The residues showing 100% Hα reincorporation are shown in red, green and yellow, 18 contacts could be predicted from those residues. Valine in pink add 6 more contacts. The predicted contacts are well distributed over the structure, showing the relevance of the Hα – Hα contribution for structure determination.

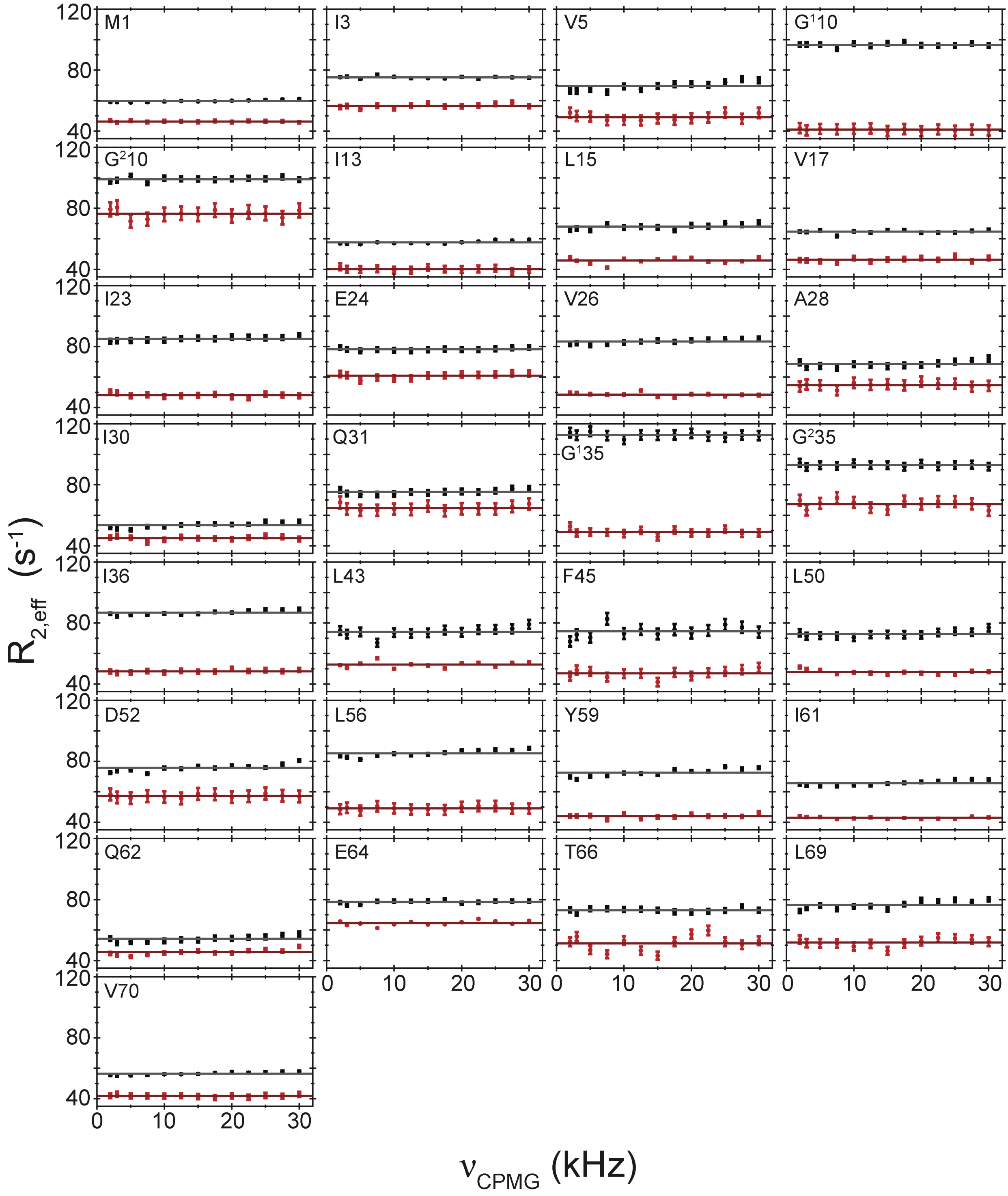

Figure S11: Transverse relaxation rates (R_2) using CPMG for fully protonated (black) and α-PET labeled ubiquitin (red). The data is from isolated peaks in the Cα-Hα HSQC of ubiquitin samples exchanged in 100% D2O at 277 K and measured at a 600 MHz spectrometer.

Residue	Transverse relaxation rate at 277 K (s^{-1})	
	^{15}N, ^{13}C ubiquitin	α-PET ubiquitin
M1	59.7 ± 0.7	46.3 ± 0.4
I3	75.2 ± 0.8	56.5 ± 1.2
V5	69.4 ± 2.8	49.0 ± 1.9
$G^1$10	96.5 ± 1.1	40.9 ± 0.7
$G^2$10	99.1 ± 1.1	76.4 ± 2.7
I13	57.6 ± 1.1	40.1 ± 0.8
L15	68.0 ± 1.6	45.7 ± 2.0
V17	64.6 ± 1.1	46.3 ± 1.2
I23	85.1 ± 1.1	48.1 ± 1.0
E24	78.2 ± 0.8	60.8 ± 1.5
V26	83.3 ± 1.4	48.5 ± 1.1
A28	68.5 ± 1.8	54.7 ± 1.4
I30	53.6 ± 1.8	45.0 ± 1.1
Q31	75.4 ± 1.3	64.8 ± 1.7
$G^1$35	112.6 ± 1.4	49.0 ± 1.7
$G^2$35	92.8 ± 0.9	67.3 ± 2.7
I36	86.8 ± 1.5	48.4 ± 0.8
L43	74.3 ± 2.7	52.7 ± 1.9
F45	74.6 ± 3.8	47.0 ± 2.5
L50	72.9 ± 1.7	47.8 ± 1.4
D52	75.7 ± 2.3	57.2 ± 1.1
L56	85.2 ± 2.1	49.2 ± 1.0
Y59	72.5 ± 2.5	44.0 ± 1.1
I61	65.7 ± 1.7	42.85 ± 0.7
Q62	54.2 ± 1.7	45.4 ± 1.7
E64	78.4 ± 1.0	64.6 ± 1.5
T66	73.0 ± 1.1	51.3 ± 5.0
L69	76.6 ± 2.6	51.8 ± 2.3
V70	56.5 ± 1.0	41.9 ± 0.6

Table S4: Transverse relaxation rates (R2), obtained from CPMG measurements at 277 K for resolved residues in uniform ^{15}N, ^{13}C labeled and α-PET labeled ubiquitin measured at a 600 MHz spectrometer.

Residue	Transverse relaxation rate at 308 K (s^{-1})	
	^{15}N, ^{13}C ubiquitin	α-PET ubiquitin
M1	21.1 ± 0.3	16.7 ± 0.5
Q2	27.3 ±0.2	24.6 ± 0.6
F4	36.5 ± 0.5	23.3 ± 0.5
V5	24.7 ± 0.8	17.3 ± 0.3
T7	25.8 ± 0.2	20.5 ± 1.9
T9	18.4 ± 0.9	17.7 ± 1.6
$G^1$10	35.7 ± 0.6	31.9 ± 1.4
$G^2$10	35.3 ± 0.3	34.0 ± 0.8
T12	32.6 ± 0.5	26.5 ± 1.1
I13	20.7 ± 0.3	15.5 ± 1.3
T14	34.0 ± 0.4	28.6 ± 1.1
L15	24.6 ± 0.9	16.8 ± 0.3
V17	24.0 ± 1.3	16.5 ± 5.0
E18	29.6 ± 0.6	18.7 ± 0.3
T22	34.1 ± 0.4	25.0 ± 1.2
I23	31.2 ± 0.6	19.5 ± 0.1
E24	28.2 ± 0.6	21.5 ± 0.3
V26	30.7 ± 0.4	19.4 ± 0.4
A28	25.4 ± 0.2	20.4 ± 0.8
I30	27.9 ± 0.2	18.9 ± 0.7
Q31	28.3 ± 0.5	23.9 ± 0.3
E34	19.9 ± 0.5	17.7 ± 0.1
$G^1$35	43.6 ± 0.4	35.0 ± 0.7
$G^2$35	43.1 ± 0.3	38.7 ± 1.5
I36	32.6 ± 0.2	18.5 ± 0.6
D39	20.2 ± 0.4	19.5 ± 0.8
Q41	27.5 ± 0.1	21.5 ± 0.7
L43	27.3 ± 0.2	19.5 ± 0.5
I44	26.2 ± 0.2	19.3 ± 0.3
F45	25.5 ± 0.2	16.8 ± 0.3
A46	18.2 ± 0.5	14.8 ± 0.9
G47	43.0 ± 0.8	39.1 ± 2.5
Q49	24.7 ± 0.3	21.1 ± 1.2
L50	26.3 ± 0.3	18.1 ± 0.2
D52	28.0 ± 0.8	21.3 ± 0.5
T55	34.7 ± 0.2	25.5 ± 0.3
L56	27.7 ± 0.1	17.3 ± 0.6
S57	24.8 ± 0.4	19.7 ± 0.7
Y59	20.6 ± 2.0	15.8 ± 2.5
N60	22.6 ± 0.2	21.5 ± 0.4
I61	24.2 ± 0.4	18.0 ± 0.4
Q62	19.4 ± 0.5	16.5 ± 0.5
34.6	32.2 ± 0.4	27.2 ± 0.4
T66	34.6 ± 0.6	27.3 ± 1.4
L67	24.6 ± 0.2	17.1 ± 0.2
L69	27.1 ± 0.4	18.7 ± 0.4
V70	20.3 ± 1.6	16.2 ± 0.4
L71	21.6 ± 0.1	15.1 ± 0.2
L73	11.1 ± 0.1	87.7 ± 0.5
G75	9.2 ± 0.1	7.6 ± 0.5

Table S5: Transverse relaxation rate (R2), obtained from CPMG measurements at 308 K for resolved residues in uniform ^{15}N, ^{13}C labeled and α-PET labeled ubiquitin measured at a 600 MHz spectrometer.

Sample	Spectrum	TD F3	TD F2	TD F1	AQ F2 (ms)	AQ F1 (ms)	NS	Total time
SH3 ref	(H)CH	*	1024	402	*	9.9	4	1h30
α-PET SH3 in H_2O	(H)CH	*	1024	402	*	9.9	4	1h30
α-PET SH3 in D_2O	(H)CH	*	1024	402	*	9.9	4	1h30
α-PET SH3 in H_2O	H(H)NH	1024	102	80	5.3 (^1H)	14.9 (^{15}N)	4	24h
α-PET SH3 in H_2O	H(H)CH	1024	160	192	4.9 (^1H)	10 (^{13}C)	2	24h
α-PET SH3 in D_2O	H(H)CH	1024	160	192	4.9 (^1H)	10 (^{13}C)	2	24h
α-PET VDAC D_20	(H)C(HH)CH	1024	294	170	4.6 (^{13}C)	4.2 (^{13}C)	2	8 d

Tabel S6: Spectrum acquisition parameters recorded for the different samples. All were recorded at a magnetic field of 18.8 T (on an 800 MHz Bruker spectrometer) at 55kHz MAS using a 3 channel narrow bore HCN probe. The temperature was set at 250K for VDAC and 260K for SH3. 10kHz of waltz16 decoupling was used for ^{13}C and ^{15}N. 12 kHz swept TPPM decoupling was used during indirect evolution periods. Water was suppressed by saturation with at 13.75 kHz for 200ms (protonated buffer) and 50ms (deutereted buffer). The spectra were processed with a squared cosine function. The direct acquisition time was 10 ms and 7 ms for SH3 and VDAC, respectively.

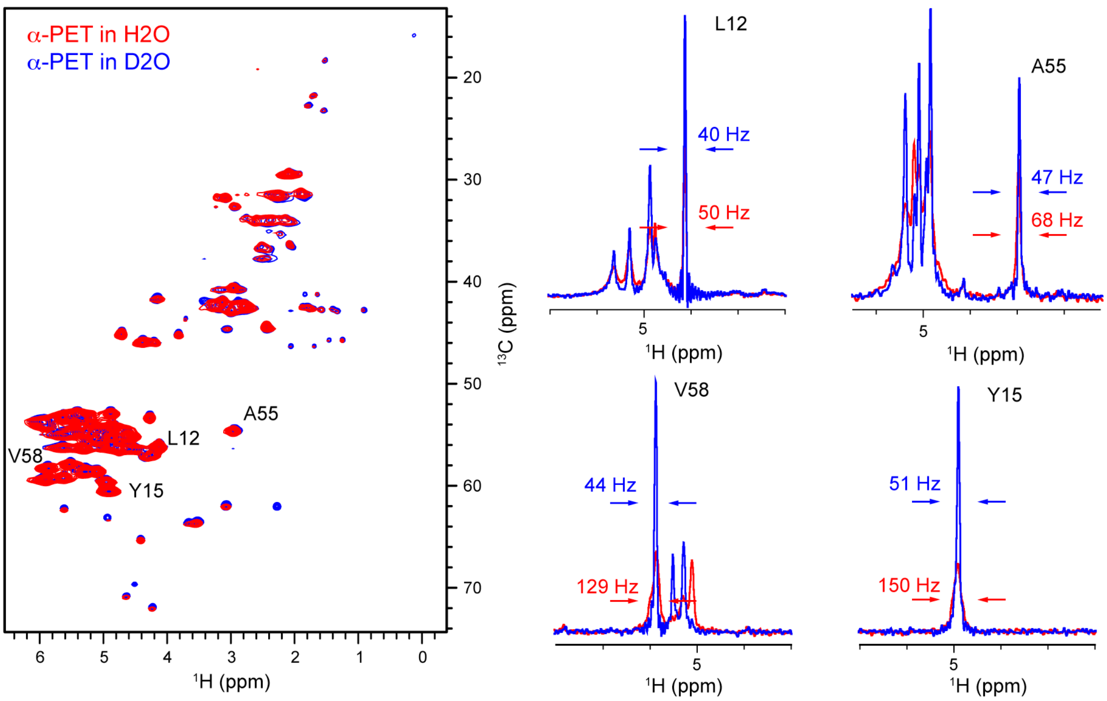

Figure S12: α-PET SH3 comparison using protonated buffer (red) or deuterated buffer (blue) for crystallization. Most of the residues show an improvement in the linewidth when amide protons are exchanged using deuterated buffer for crystallization. When linewidths are reported, no apodization was applied for the reported dimension.

[1] J. Medeiros-Silva, D. Mance, M. Daniels, S. Jekhmane, K. Houben, M. Baldus, M. Weingarth *Angew. Chem. Int. Ed. Engl.* **2016**, *55*, 13606-13610.
